# A Cognitive Computational Model Inspired by the Immune System Response

**DOI:** 10.1155/2014/852181

**Published:** 2014-06-09

**Authors:** Mohamed Abdo Abd Al-Hady, Amr Ahmed Badr, Mostafa Abd Al-Azim Mostafa

**Affiliations:** ^1^Arab Academy for Science, Technology and Maritime Transport, Faculty of Computing and Information Technology, Cairo 1029, Egypt; ^2^Faculty of Computers and Information, Cairo University, Cairo, Egypt

## Abstract

The immune system has a cognitive ability to differentiate between healthy and unhealthy cells. The immune system response (ISR) is stimulated by a disorder in the temporary fuzzy state that is oscillating between the healthy and unhealthy states. However, modeling the immune system is an enormous challenge; the paper introduces an extensive summary of how the immune system response functions, as an overview of a complex topic, to present the immune system as a cognitive intelligent agent. The homogeneity and perfection of the natural immune system have been always standing out as the sought-after model we attempted to imitate while building our proposed model of cognitive architecture. The paper divides the ISR into four logical phases: setting a computational architectural diagram for each phase, proceeding from functional perspectives (input, process, and output), and their consequences. The proposed architecture components are defined by matching biological operations with computational functions and hence with the framework of the paper. On the other hand, the architecture focuses on the interoperability of main theoretical immunological perspectives (classic, cognitive, and danger theory), as related to computer science terminologies. The paper presents a descriptive model of immune system, to figure out the nature of response, deemed to be intrinsic for building a hybrid computational model based on a cognitive intelligent agent perspective and inspired by the natural biology. To that end, this paper highlights the ISR phases as applied to a case study on hepatitis C virus, meanwhile illustrating our proposed architecture perspective.

## 1. Introduction

The immune system (IS) is by nature a highly distributed, adaptive, and self-organized system that maintains a memory of past encounters and has the ability to continuously learn about new encounters; the immune system as a whole is being interpreted as an intelligent agent. The immune system, along with the central nervous system, represents the most complex biological system in nature [1]. This paper is an attempt to investigate and analyze the immune system response (ISR) in an effort to build a framework inspired by ISR. This framework maintains the same features as the IS itself; it is cognitive, adaptive, fault-tolerant, and fuzzy conceptually. The paper sets three phases for ISR operating sequentially, namely, “recognition,” “decision making,” and “execution,” in addition to another phase operating in parallel which is “maturation.” This paper approaches these phases in detail as a component based architecture model. Then, we will introduce a proposal for a new hybrid and cognitive architecture inspired by ISR. The framework could be used in interdisciplinary systems as manifested in the ISR simulation. Then we will be moving to a high level architecture for the complex adaptive system. IS, as a first class adaptive system, operates on the body context (antigens, body cells, and immune cells). ISR matured over time and enriched its own knowledge base, while neither the context nor the knowledge base is constant, so the response will not be exactly the same even when the immune system encounters the same antigen. A wide range of disciplines is to be discussed in the paper, including artificial intelligence, computational immunology, artificial immune system, and distributed complex adaptive systems. Immunology is one of the fields in biology where the roles of computational and mathematical modeling and analysis were recognized [1].

The paper supposes that immune system is a cognitive system; IS has beliefs, knowledge, and view about concrete things in our bodies, which gives IS the ability to abstract, filter, and classify the information to take the proper decisions.

The paper targets a host of objectives including building a computational model for ISR along with a deep analysis for ISR and the operations involved. This approach aims to advance the researches based on integrative empirical data emanating from independent sources, as well as having its influence on researchers seeking to make novel predictions regarding the immune system response to infectious diseases. The proposed architecture furthermore could be helpful in developing a simulation tool for studying the interactions between pathogens and hosts' IS [2].

The paper discussion touches upon seven perspectives: first, “strategy” which indicates what to do in which circumstances; second, “agent” which is a collection of properties strategies and capabilities for interacting with artifacts and other agents; third, “variety” which is the diversity of types within a population or system; fourth, “interaction pattern” by which we mean the recurring regularities of contact among types within a system; fifth, “location” which is a set of categories structured so that nearby agents will tend to interact; sixth, “selection” which refers to the processes that lead to an increase or decrease in the frequency of various types of agents or strategies; and seventh, “success” which is criteria or performance measures used by an agent in attributing credit in the selection of relatively successful or unsuccessful strategies or agents [3].

## 2. Problem Description

The big picture of ISR is still undefined and a cognitive computational model for ISR operations is not defined as integrated system, since defining a computational model for a part of immune system operations will not cover the perfection of a multidisciplinary integrative system like the natural biological immune system. Immunology needs precise mathematical modeling and computer simulation to help us understand the emergence of immune specificity from the collective coresponse. The interactions are simply too complex to be grasped by intuition [4]. Computational modeling of the immune system can support practical applications, particularly the demonstrated ability to accelerate discovery through simulation driven experimentation [1].

Understanding the nature of ISR which is complex, distributed, adaptive, and intelligent and identifying the components of the system are two enigmatic tasks, because some ISR are by nature poorly understood [5]. Classic, cognitive, and danger theories are the most common and valid ISR theories adopted by immunology scientist community. However each theory concentrates on one point of view and ignores some logical consequences. After deep analysis, and though for ISR from perspective of behavioral aspect-oriented, we found that, building a cognitive model will rely upon the existing theories as complementary for the big picture. Since most related work describes ISR from one perspective, it does not accord with the real complex nature of the natural biological system and the results of its computations and operations. Researchers have tackled the interdisciplinary ISR from specialized narrow perspectives, coming up with a fragmented conception of how the ISR is likely to be as a whole. Disciplines involve distributed computation, fault tolerance, dynamic learning, adaptation, and self-monitoring with a solid biological knowledge [6].

The proposed view provides an attempt to draw an inclusive function-oriented picture of the entire components. This picture is represented by a diagram in which each object stands for a “component” that takes inputs and returns outputs after processing, which is the traditional model of program execution, and could be implemented using current software paradigms, for example, function-oriented, object-oriented, dynamic-oriented, and agent-oriented tools and languages.

The underlying proposition of the paper is to try to provide an answer for the questions: “why does the immune system respond in different ways to different situations?” and “is immune system a cognitive system?”

## 3. Discussion

### 3.1. High Level Immune System Phases

**Figure 1:**
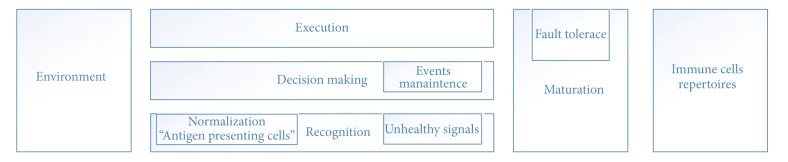
High level immune system phases.

ISR can be logically divided into three phases: the first has to do with antigen recognition; the second is related to decision making; and the third phase is related to action execution (see Figure 1). ISR is able to answer such questions as how IS eliminates the invading pathogen and how IS puts the response plan into effect.

The response implicates saving plasma memory cells for quick ISR as well as overall system self-organization. However, maturation is a continuous process starting with naive cells, which remain sensitive for recognition, decision making, and actions execution.

### 3.2. Maturation Phase

**Figure 2:**
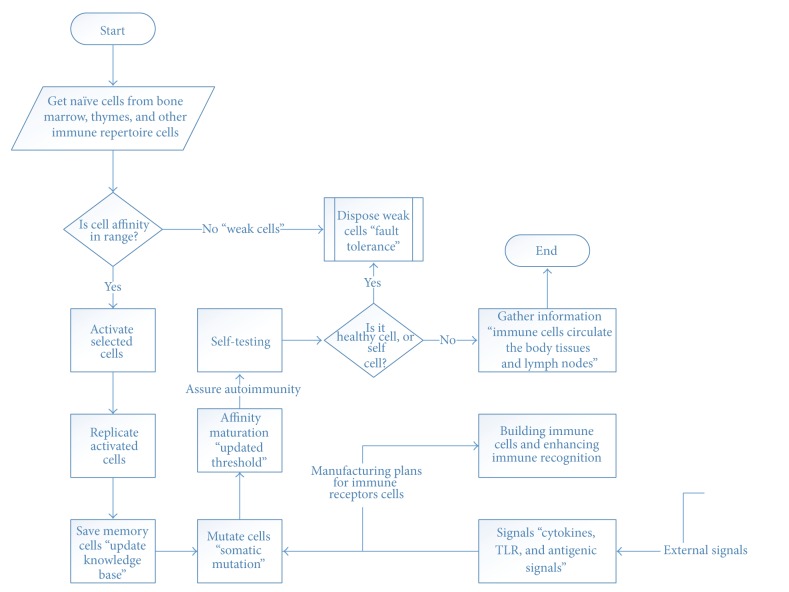
Maturation phase.

Maturation is a continuing phase of enhancing self-experience, adaptation, and optimizing internal operations over time (see Figure 2).

(1) Immune system cells originate in the bone marrow; precursor cells generated in the bone marrow develop into all the cellular elements of blood including those of the immune system. As they age, precursor cells differentiate into specialized cells. Lymphoid cells differentiate into the B cells and T cells of the adaptive immune system, known as lymphocytes and the natural killer cells as a part of innate immune system. When T lymphocytes leave the bone marrow, they have not yet matured. They migrate through the lymphatic system into the thymus (an organ located near the heart). In the thymus, T cells undergo further testing, reduction, and filtering with roughly 2–4% developing into mature T cells. B cells also undergo this testing and filtering but in the bone marrow [5].

(2) Immature B cells are tested for autoreactivity before they leave the bone marrow by present young lymphocytes with self-antigens produced throughout the body and to eliminate those cells that recognize self-antigens and preventing autoimmunity. Antigen recognition in the absence of costimulation leads to functional inactivation and activating naive T cells or clonal deletion of peripheral T cells [5]. To prevent autoimmunity, the differentiation of lymphocytes is accompanied by selective mechanisms that ensure tolerance to self-components through the destruction or the inactivation of autoreactive clones [1].

(3) While the bone marrow produces fresh immune cells, the thymus selects those cells that are immunocompetent and are allowed to enter the body. The blood lymph vessels and mature immune cells circulate between the body tissues and the lymph nodes on their way through the tissues', immune cells gather information on the tissues' state, the presence of antigens, and the activity of other immune cells. This information is available through abstract molecular shapes that are presented by all antigens and tissue cells [7].

(4) The first selection step takes place during the maturation (immune has to mutate its cell and the mutation rate is increased by antigen stimulation [5]) process in the thymus where the new immune cells move after being produced in the bone marrow. In general, only those cells surviving these evolutionary steps are able to bind molecules that are derived from the self of the body (healthy cells). Thus, the cell's ability to recognize the organism's molecules is tested.

(5) The affinity between receptor and tested molecule is neither allowed to be too strong nor allowed to be too weak; only moderately binding cells survive and enter the circulation. All other cells are deleted. In case of high affinity the cells are selected and become active [7].

(6) Activated cells are replicated into plasma cells “memory cells”; immune system uses its memory cell for fast response to predefined antigen/pathogen. Because the total amount of available immune cells in the body is (in terms of binding epitopes) smaller than the potentially dangerous molecules (pathogens), cells that becomes activated by a successful recognition event reproduces itself and inherits its receptor genes to its offspring. However, the daughter cells somatically modify the receptor genes once again; this process is called hypermutation. Hypermutation allows for the selection of B cells that express immunoglobulin receptors possessing an enhanced ability to recognize and bind a specific foreign antigen [5].

(7) The receptors of the lymphocytes are created somatically, which means immune cells can manufacture their receptors epigenetically from genetic raw material [5]. Each developing lymphocyte generates a unique antigen receptor by rearranging its receptor gene segments. While a receptor's reaction site is genetically determined by the germ-line of the organism, thus it is limited to a few possible shapes. The genes of a receptor's binding site are composed individually by rearranging short pieces of DNA. After that, a random sequence of mutation, deletion, and addition operators is applied to the segments of the receptor's genetic “building plan” [7], and the development and survival of lymphocytes are determined by signals received through their antigen receptors [5].

(8) The term affinity describes the specific binding energy between receptor and ligands that arises from their degree of molecular complementary; the higher affinity is the higher probability of a successful binding event [7].

(9) Affinity maturation is the process by which immune cells produce antibodies with increased affinity for antigen during an ISR. Due to repeated exposures to the same antigen, a host will produce antibodies of successively greater affinities [8].

(10) Affinity measures determine the degree to which the immune system differentiates between different antigens. Cross-reactivity measures the extent to which different antigens appear similar to the immune system. The molecular determinants of specificity and cross-reactivity define the nature of antigenic variation and the selective processes that shape the distribution of variants in populations [9]. Updates on affinity measures affect the threshold of immune system stimulation and sensing antigenic exposure.

(11) The matured antibody had an affinity for the epitope 30,000 times higher than the original naive antibody [10].

(12) Immune system achieves self-tolerance by eliminating those T cells and B cells that react to self when lymphocytes undergo maturation on thymus and bone marrow. In addition, a “confirmation” costimulation signal is required; that is, for either B cell or T natural killer cell activation, a T helper lymphocyte must also be activated. This dual activation is a further protection against the chance of accidentally reacting to self [11]; that is, when the peptide major histocompatibility complex (MHC) is presented on the surface of the cell, it might bind to a CD8 T cell with a fitting T cell receptor (TCR), however such a TCR clone exists depend on among other factors, e.g., when the TCR-peptide complex is too similar to MHC-peptide complexes generated with peptides from the host self-peptides, this effect is called tolerance and might be broken by so-called self-epitopes [12].

(13) Clonal energy: when T cells need two signals to be activated, one signal from the TCR-binding antigen/MHC, and co-stimulus provided by the antigen presenting cell. If the T cell receives signal one alone, it is rendered anergic and cannot respond to a subsequent encounter with the same antigen even if it receives the costimulus. Regulatory cells TR are able to stop self-reactive CD4 T cells responding even if the CD4 T cell receives the two signals required for activation [13].

(14) Clonal ignorance: inevitably antigen receptor production leads to development of autoreactive receptors, therefore increasing of self-antigens binding leads to programmed cell death apoptosis [14]; this is considered as a mechanism used by immune system to grant self-tolerant feature.

### 3.3. Recognition Phase

**Figure 3:**
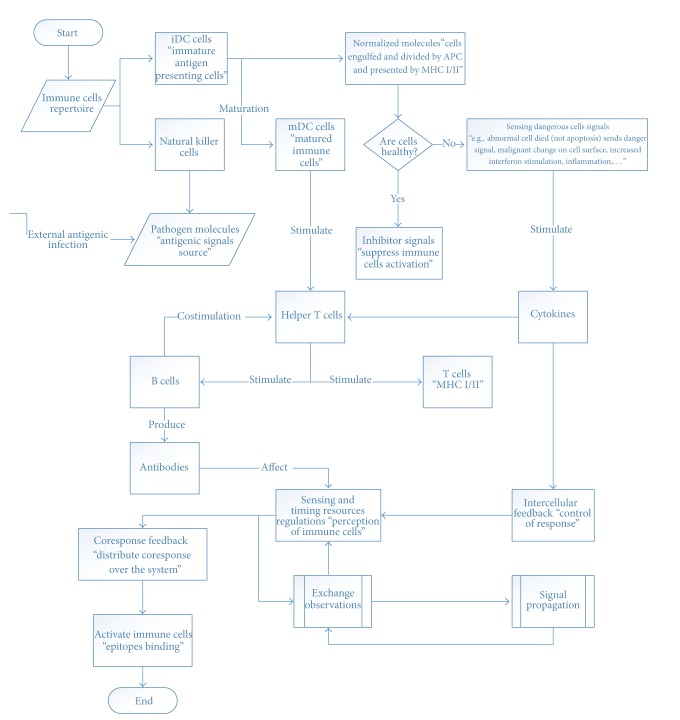
Recognition phase.

(1) Immune cells filter out the relevant molecular signals from the total set of signals, which are available in the body. The ability to discriminate between relevant and irrelevant signals is called the specificity of recognition [7] (see Figure 3).

(2) Immune cells focus on different aspect of the antigenic world in the recognition phase; a distributed multilevel perception is achieved that yields a detailed image of the whole body's state. This molecular image is the starting point for the cellular interactions of the next phase where an appropriate ISR is selected [7].

(3) Immature dendritic cells (iDCs) migrate through the bloodstream from the bone marrow to enter tissues and triggered to activate naive T lymphocytes. The molecules recognized by pattern recognition receptors are quite distinct from the individual pathogen specific antigens recognized by lymphocytes. The fact that microbial constituents were needed to stimulate ISR against purified proteins highlights the requirement that an innate response must precede the initiation of an adaptive response [5].

(4) Macrophages are scavenger cells that can be induced by pathogens to present foreign antigens to naive T cells [5].

(5) B cells are highly efficient at presenting antigens that bind to their surface “immunoglobulin” and are activated by helper T cells that recognize the same antigen [5].

(6) Macrophages and dendritic cells (DCs) activated by pathogens secrete a range of cytokines that have a variety of local and distant effects [5]. Immature dendritic cells are very active in ingesting antigens by phagocytosis using complement receptors, which recognize the constant regions of antibodies in antigen antibody complexes.

(7) Immature dendritic cells are exposed to pathogens leading to activation of their toll-like receptors (TLRs). TLR signaling causes the dendritic cells to become licensed and begin to undergo maturation, which involves induction of the chemokine receptor CCR7. TLR signaling also increases the processing of antigens. In addition to the display of antigens that activates the antigen-receptors of lymphocytes, mature dendritic cells also express cell-surface proteins called costimulatory molecules, which provide signals that act together with antigen to stimulate the T lymphocyte to proliferate and differentiate into its final fully functional form [5].

(8) Immune system performs continuous processes of sensing dangerous signals, for example, intracellular signals and apoptosis (programmed cell death signals). Cell stimulates special cytokines differentiated by immune system sensing process, when cell was destroyed by invading antigen. According to the danger theory, a cell that dies unnaturally sends out the danger/alarm signal and the danger signal establishes a danger zone around itself [15]. On the other hand, the antigens near the cell that emits the danger signal are captured by APCs such as macrophages and then travel to the local lymph node and present the antigens to lymphocytes [16].

(9) Specific recognition of dead and dying cells is essential and during apoptosis a loss of “do not eat me” signals and a gain of “eat me” signals, will stimulate phagocytes cells. However, relatively little is known about the identity and structure of the apoptotic cell. The most characterized feature of these specific surface changes is generating a negative charge at the cell surface and this can mediate clearance of apoptotic cell and exposure of intracellular components [17], for example, the antibodies which cover the cell surface acting as tags “marking” it as foreign; now any phagocytic cell like a macrophage will engulf the antigen and destroy it [18]. Also at the molecular level, MHC proteins present peptide antigens on cell surfaces peptides, where cleaved peptides antigen proteins are integral to the process of antigen recognition by cytotoxic and helper T cells, whereas soluble cytokines are crucial for intercellular signaling. At the cellular level, cytotoxic T cells contribute to the neutralization of intracellular pathogens and potential cancers by eliminating the infected or malfunctioning cells, whereas plasma cells derived from B cells contribute to the neutralization of extracellular pathogens through the production of antibodies. At the organ level, the thymus has an essential role in the maturation of T cells and the elimination of self-reactive T cells, while the lymphatic system provides an essential mechanism for transporting ISR cells and molecules to sites of infection [18].

(10) Natural killer NK cells are activated by interferon and macrophage derived cytokines to serve as an early defense against certain intracellular infections; also NK cells possess receptors for self-molecules that prevent their activation by uninfected cells [5].

(11) T cell receptor recognizes antigen in the form of a complex of a foreign peptide bound to an MHC I (MHC class I molecules bind short peptides of 8 to 10 amino acids by both ends)/II (the length of the peptides bound by MHC class II molecules is not constrained). The MHC I/II molecules deliver peptides to the cell surface from two intracellular compartments and specialized MHC class I molecules act as ligands for activation and inhibition of NK cells [5].

(12) T cells, B cells, and macrophages use immune molecules to communicate their response to each other and other tissues of the body. This forms an immune dialogue comprised of an ongoing exchange of chemical signals between the immune cells. Subject to this exchange of information, they update their own responses accordingly, be it to increase or decrease the vigor of their response [19].

(13) Intracellular signal propagation is mediated by large multiprotein signaling complexes. APC has three signals. “Signal one” is delivered through the T cell receptor, when it engages an appropriate peptide MHC. Signal one alone is thought to promote naive T cell inactivation by anergy deletion or cooption into a regulatory cell fate, thereby leading to tolerance. “Signal two” is referred to as cosimulation and is taken to mean an accessory signal(s) that together with signal one induces immunity. This is often measured as T cell clonal expansion differentiation into effectors cells and a long-term increase in precursor frequency memory; however the actual signal two is a fine balance of positive and negative costimulatory signals emanating from many receptors. “Signal three” refers to signals delivered from the APC to the T cell that determine its differentiation into an effectors' cell, for example, differentiation into T helper (TH1) cells, TH2 cells, or cytotoxic T lymphocytes (CTLs) [20].

(14) Antibodies protect against extracellular pathogens and their toxic products and bind a wide variety of chemical structures, in addition to binding to conformational shapes on the surfaces of antigens. The constant region confers functional specialization on the antibody [5].

(15) The rule engine is the essential mediator of correspondents as a decision set to detect, infer, and react to incoming events and process event patterns [21], and from immune system perspectives, ISR is a result of triggered events (T helper cells trigger immune cells' reaction and activation for both B cell and T cell and epitopes binding will trigger cell inhibitors or activate other immune cells) and will lead to continues body feeds (cytokines); then, the response is matured by knowledge base [14]. The fact that each immune cell bears receptors, that collected as input part of the output of the other immune cells. Thus, each cell sees what it sees of the body's infection, while it also sees the effect on other immune cells of their own perceptions of the infection. In fact, there are classes of immune cells regulatory cells that specialize in responding not to the states of body cells, but directly to the states of other immune cells. Integration of the resulting inflammatory response takes place because each cell updates its own output in coresponse to the output of its fellow cells. In other words each immune cell participates in the collective regulation of the inflammatory response that maintains the organism [22]. So the immune regulation or the ability of the immune system to self-regulate is thus an important feature of ISR and failure of such regulation contributes to conditions such as allergy and autoimmune disease [5].

### 3.4. Decision Making Phase

**Figure 4:**
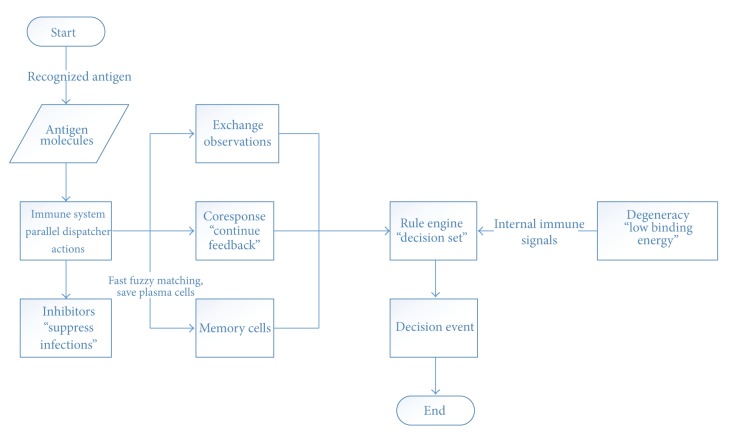
Decision making phase.

After the immune cells have gathered the molecular signals in the tissues of the body, they move to the lymph nodes and mutually exchange their observations. The cells react in accordance with their recent perceptions and as a result influence the reactions of other cells, and thus the ISR is a coherent system [7] (see Figure 4).

(1) Cytokines molecules (intercellular feedback mechanism) can have stimulating and inhibiting effects on the immune cells, and effects can even change in the course of an ISR and so they are an important factor in setting off and influencing the correspondents [7].

(2) Memory cells that are a result of a successful maintenance event took place in the past. These cells proved to be efficient in causing an ISR and therefore changed to this cellular memory state. So in case of repeatedly occurring antigens, the memory cells can speed up the necessary immune decision and reproduce an efficient response to the antigen more quickly [7].

(3) Inhibitory receptors, which dampen cellular activation, play the dominant role whereas every NK cell has at least one inhibitory receptor. By default, the NK cell will be active by the same principle that is if NK cells inhibitory receptor(s) is not engaged, the NK cell will be activated [23].

(4) Degeneracy is property of a receptor that is able to bind more than one single ligand (V region of epitopes); that is, an immune cell receptor corresponds to a general key that also opens locks with similar shapes and not just the one with the exact complementary shape. This flexible mapping between receptors and ligands is called degeneracy [4], which means a low binding energy will be signaled.

(5) Rule engine is a decision set to detect, infer, and react to incoming events and process event patterns [21], and from immune system perspectives, ISR is a result of triggered events (T helper cells trigger immune cells' reaction and activation for both B cell and T cell and epitopes binding will trigger cell inhibitors or activate other immune cells) and will lead to continues body feeds (cytokines); then, the response is matured by knowledge base [14].

### 3.5. Execution Phase

**Figure 5:**
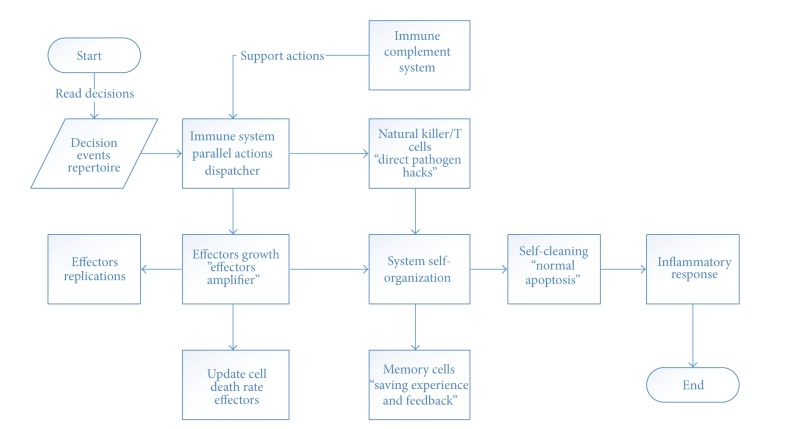
Execution phase.

The immune cells spread across the body tissues once more and execute the decision that they have mutually come; despite the degenerate perceptions of the cells, the immune system shows the ability to respond specifically [7] (see Figure 5).

(1) Dendritic cells initiate and regulate the pathogen specific adaptive ISR and thus are central to the development of adaptive ISR as well as immune tolerance [24].

(2) The complement system is a group of proteins, which recognizes features of microbial surfaces and marks them for destruction by the deposition [5]. Antibodies often combine with complement proteins activating the complement proteins to produce lesions in the antigenic membrane complement proteins, or antibodies will attach to foreign cells and thereby stimulate phagocytes to ingest those cells, which is called opsonization [25].

(3) Effectors cells grow based on recruitment of ISR and proliferation, which depends on cell death rate as reproduction rate [11]. Effectors cells start with a certain concentration value and by the time this value is decreased [26], effectors cell types depend on the nature of the signals they receive during priming [5].

(4) Once the cells have differentiated into effectors T cells, any encounter with specific antigen triggers their effectors actions without the need for costimulation. This distinction is particularly easy to understand for CD8 cytotoxic T cells, which must be able to act on any cell infected with a virus, whether the infected cell can express costimulatory molecules. However, this feature is also important for the effectors function of CD4 cells. Effectors CD4 T cells must be able to activate B cells and macrophages that have taken up antigen, even if these cells are not initially expressing costimulatory molecules [5].

(5) NK cells will start attacking immediately; the attack will be either direct if the targeted cell is an infected cell or indirect if the targeted cell is an antigen via a specific antibody. On cell attack, the interaction takes the form of toxin injection from the NK into infected cell and is usually lethal on response regulation and the interaction amounts to cytosine injection from the NK into the bloodstream; this either suppresses or stimulates the development of T-lymphocytes, each of which may inherit different features, therefore stimulate other immune cells response and may stimulate regulate NK proliferation [27].

(6) B memory cell is involved in the regulation of the ISR. After the reception of cytosines, a B memory cell will in turn inject cytosines into the bloodstream. This will result in a number of interactions whereby the cell may either proliferate or suppress the immune activity [27].

(7) Immune regulation is the fact that each immune cell bears receptors that collect as input part of the output of the other immune cells. Thus, each cell sees what it sees of the body's injury, while it also sees the effect on other immune cells of their own perceptions of the injury. In fact, there are classes of immune cells (regulatory cells) that specialize in responding not to the states of body cells, but directly to the states of other immune cells. Integration of the resulting inflammatory response takes place because each cell updates its own output in coresponse to the output of its fellow cells. In other words, each immune cell participates in the collective regulation of the inflammatory response that maintains the organism [22].

(8) Inhibitory receptors on lymphocytes help regulate ISR; cytokine signaling is terminated by a negative feedback mechanism. The receptors that induce apoptosis activate specialized intracellular proteases called caspases [5].

(9) Effectors' cells that divided the maximum number of times stop dividing and wait for apoptosis based on cell effectors type death rate [28].

(10) Various patterns of cytokine secretion are seen depending on the mode of injury leading to the inflammatory response [29].

(11) Immune system is self-organized, because the total amount of available immune cells in the body is smaller than the potentially dangerous molecules (pathogens); the cells' perceptions are focused; that is, certain lymphocyte B cells sense the molecular shapes of antigens directly, while other lymphocytes T cells need the assistance of monocytes that present preprocessed antigen molecules to them. Immune cells focus on different aspects of the antigenic world in the recognition phase. Distributed multilevel perception is achieved that yields a detailed image of the whole body's state. This molecular image is the starting point for the cellular interactions of the next phase where an appropriate ISR is selected [7].

(12) Immune system self-tolerance was discussed earlier on maturation phase.

(13) Inflammation is one of the first responses of the immune system to infection [30]. Inflammation turn triggers a response of body cells in the area of injury leading usually to healing and restoration of functions. As process evolves, the immune system updates the inflammatory response to match the particular circumstances, which emerge on the way to healing, maintaining, and/or defending the body. The output of the immune system is the healing process (the inflammatory response) that maintains a healthy body [22].

(14) The nature of an inflammatory response depends on a continuous computation based on the collective interactions between immune and body cells. These interactions are required throughout one's lifetime; only upon death does the immune system terminate its computations of the state of the body. The bottom line is that the immune system is a continuously reactive computing system [22].

### 3.6. Hybrid Modeling for Immune System and Intelligent Agent

**Figure 6:**
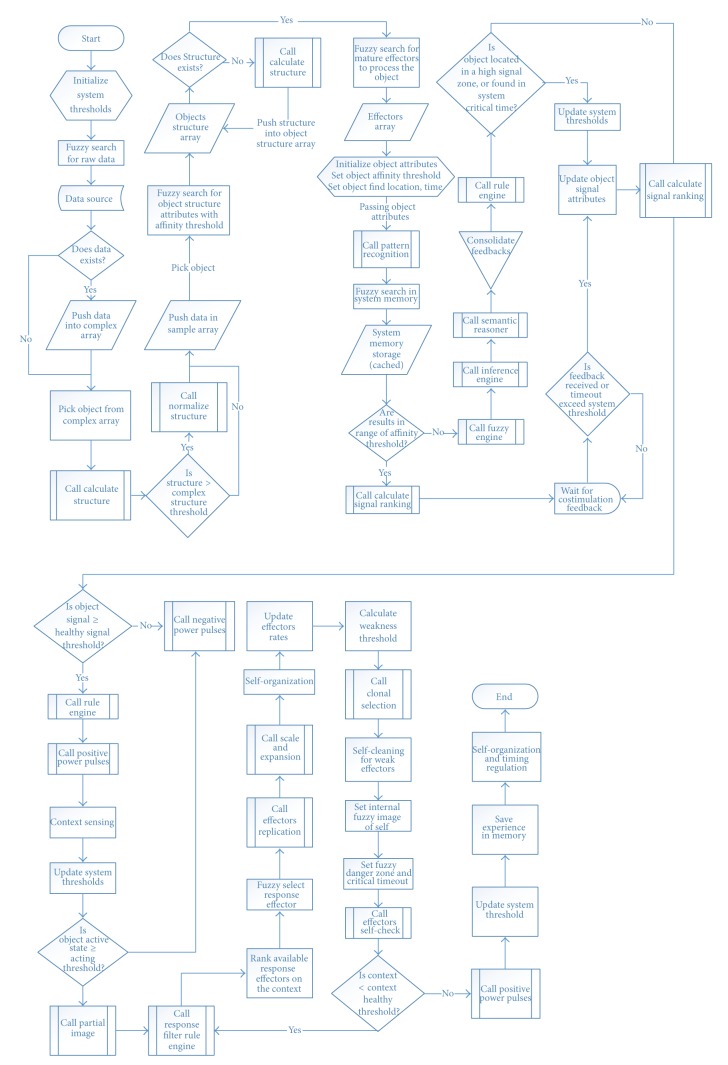
Hybrid computational model.

The immune system works as an integrated whole intelligent system and runs in adaptive workflow (see Figure 6). System can respond potentially in many ways and even contradictory ways. We will discuss how the proposal could help both better modelling and better comprehension of the immune system machinery. When IS detects an injury or an antigen, the outcome of any ISR involves a choice between many alternative types of possible response, and different types of cells take part in the response choice. This immune decision making process uses strategies similar to those observed in nervous system cognition [22].

The proposed hybrid model comes with three parts: services, helpers, and core system. Services are component/function based architecture that gets inputs and does processing and then returns the output to the caller or cooperates with another service. Helpers are functional based architecture, for example, “self-checking, context sensing, system maturation, cleaning and tolerance support, scale and expansion unit, and effectors rates (growth, death,…).” Core system processes the adaptive workflow and manages and controls the integrate processes.

#### 3.6.1. Core System

(1) Data normalization module: the initial process divides large and complex data inputs into smaller and simple form. APCs on immune system digest the antigenic molecules into fragments of 5–15 amino acids [13]. Phagocytes generally patrol the body searching for pathogens but can be called to specific locations by cytokines [31]. Processing simple form data enhances overall system operations and in particular pattern recognition operation.

(2) Signal/noise filter module: “how to focus recognition?” Process system inputs and performs data filtration operations to select interesting inputs. Immune system APCs can recognize a vast array of amino acids and protein conformations. A single molecule may have many antigenic determinants; the immune system must filter out those noncritical (to survival) determinants and focus on those that are hazardous [32].

(3) Context filter module: “when to act?” As per danger theory, not all nonself molecules are harmful molecules and not all self-molecules are healthy molecules; also not all antigenic signals require ISR [33]; however, usually ISR requires costimulation [5]. A quantitative representation of change on a cell metabolic state by external stimulus could be computed by tunable activation threshold model [34]. The current context provides information whether the antigenic determinant is a hazard or is harmless [32]. This service provides additional information to the filtering process as when to attack and when not to do so.

(4) Self-organization and timing regulation module: the ability of the immune system to self-regulation is an important feature of ISR, and failure of such regulation contributes to conditions such as allergy and autoimmune disease [5]. The influence of different affinities among interacting functional units leads to self-organizing properties [35]. This module has to get quantified answer for the questions of “how at the intracellular level signal transduction sensibly negotiates among all these signals,” although many of which are contradictory and simultaneous [36]. Self-organization is also responsible for replacing the less functional (weak) immune cells or apoptosis cells, with a new cells (mature/immature cells), it's the operation of destruction and construction.

(5) Internal imaging module: immune and nervous systems influence each other's activities. Homunculus is encoded in groups of neurons of the central nervous system based on observations from cognitive neuroscience such as phantom limbs; neurological and immunological homunculus are a reduced virtual image of the bodies which implements a theory of internal image of self, this theory claims that the immune system encodes a mirror image of the self-molecules in self reacting lymphocytes, providing a theory to support natural autoimmunity. This image of self-molecules is skewed in that it consists of a set of dominant self-antigens. Cohen proposes that autoimmunity is selected for by evolution, that it is kept in check by regulatory networks, and that it comes into effect to fight tumors and microbial antigens that have self-like receptors [32].

(6) Memory module: memory and self-learning are keys of body protection. When facing a new unknown antigen not only will the immune system battle the invader, but also it will learn the invader structure called unfolding. As a result of antigen unfolding, the immune system will save antigen signature in memory cells and will evolve a collection of lymphocytes specially designed and designated to detect and protect the body against the invaders [27].

(7) Basically all effective vaccines engage directly with and are subsequently recognized by effectors of immune memory [37].

(8) The response filter module: it answers the question of how to choose the most suitable immune effect [32].

(9) The response effectors module: effectors are the immune agents that are able to interact with other objects in their surrounding, and the closest objects are the most affected by [7]. All messages directed to the effectors are executed and effectors control the actions of the system on and within the body [38], by rapidly deploying resources to wherever they are needed [32]. Effectors respond by binding to antigen and facilitating its elimination [11]. After the effectors have performed the actions, the environment delivers a feedback about the agent's behavior. This feedback affects overall immune behavior and represents current body state [7].

#### 3.6.2. Services

(1) Pattern recognition service: “what can the system see?” Immune cells receptors recognize antigens peptides and mature its cells for enhancing the recognition ability. Recognition is not a Boolean operation; it is a function of the quantity, quality, timing, and location of the event. What the system can do is based upon the repertoire of receptors and what the system can see [32].

(2) Fuzzy engine service: it implements fuzzy logic (fuzzy systems become handy when someone intends to work with vague, ambiguous, imprecise, noisy, or missing information). System matches with its environment through fuzzy engine with adaptive rule base and can therefore be considered as a type of intelligent agent.

(3) Semantic reasoner engine service: it infers logical consequences from a set of facts or beliefs and uses forward chaining and backward chaining.

(4) Inference engine service: it derives answers from a knowledge base. It is the “brain” that the system uses to reason about the information in the knowledge base for the ultimate purpose of formulating new conclusions.

(5) Signal ranking service: the body cells and immune cells are producing signals (usually signal occurs when binding exceeds the affinity threshold; there is some chance that it is a false signal autoimmune reaction [39]) for molecules that call particular immune cells to sites of interaction. The cells have many thousands of receptors simultaneously gathering a large amount of diverse input from both outside and inside the cell. These receptors generate signals within the cell that become integrated by intracellular signal-transduction networks, leading to the dynamic activation of genes or to the silencing of genes [39, 40]. While the danger signal establishes a danger zone around itself, outside this danger zone, immune cells has very less probability to be stimulated. Matzinger admits that the exact nature of the danger signal is unclear; it may be a “positive” signal, for example, heat shock protein release, or a “negative” signal, for example, lack of synaptic contact with a dendritic antigen presenting cell [11]. The immune system integrates these signals at the cellular level and continuously updates its activities; however immature cells are unable to accept costimulation signal from any source [16]. The immune system is a reactive system just like a dialogue; the molecules create a reactive mechanism by which the immune system makes its judgments [36]. Signal ranking service operates on signals to grant rank for each signal as a service for signal noise filter module.

(6) Rule engine service: system agents/defectors have a set of rules, to be validate and to apply the consequences actions, The major rules applied by immune system to define danger zone, are as follows [15].

- Law 1: two signals are needed to activate the lymphocyte and the lymphocyte will die if it receives “signal one” without the costimulation of signal two; in the absence of signal one, signal two will be ignored.
- Law 2: signal one can come from any cell; however signal two comes from APCs, and the activation for B cell comes from T helper cells.
- Law 3: activated (effectors) cells do not need signal two, which revert to resting state after a short time. Immature cells are unable to accept signal two from any source [16].
- Law 4: the two-signal model takes into account danger model, whereas the lymphocytes need two signals to become activated. Costimulation is a signal that means “this antigen is really dangerous” [33].

#### 3.6.3. Helpers

(1) Clonal selection helper module: effective immune responding requires costimulated cells become activated and proliferate (replicated). Immune cells learn and adapt to the patterns presented to them in the form of pathogens [41]. Clonal selection theory suggests that autoimmunity is the result of leaks in the maturation process, where antigen receptors for self, are evade from negative selection [32].

(2) Self-checking helper module: phagocytes cells ingest T cells into peptides, meanwhile phagocytes are by nature cells; this means phagocytes cells check other phagocytes for antigenic presentation. As per Cohen comments that autoimmunity is not a defect rather a property of all healthy immune systems, therefore some autoimmunity is highly structured and predictable, these observations regarding autoimmunity were used as the basis of Cohen's immunological homunculus (immunculus), which suggests that self-reacting receptors are kept in check by regulatory networks and that such receptors respond in an altered context [32].

(3) Scales and expansion helper module: It is observable that the speed of the immune response is a result of signal amplification; that occurs followed by sequential cellular enzymes' activation of molecules complement, which in turn activates other complement enzymes and so on. This produces a catalytic (increasing rate of chemical reaction, due to participation of substance) cascade that amplifies the initial signal by controlled positive feedback [42]. The cell's many thousands of receptors simultaneously gather a large amount of diverse input from both outside and inside the cell; these receptors generate signals within the cell that become integrated by intracellular signal-transduction networks, leading to the dynamic activation of genes or to the silencing of genes changes in the shape and movements of the cell [22].

(4) Partial imaging module: each immune cell sees part of picture and transmits the signal for what it sees [22], while immune system maintains the body by deploying a reduced virtual image of the body (homunculus) represented in the molecular inputs and outputs of organized immune system cells and includes the innate receptors that also receive input from body molecules [22, 32].

(5) Parallel and distributed processing module: immune system has the following features as a distributed system [32].

- The immune system of a human is composed of many millions of individual cells, each of which is an individual processor. The computation emerges from the integration of these processors working in parallel; the integration occurs through networking.
- Each cell by its thousands of receptors collects input and each cell by its secretions and behaviors translates input into output.
- The networking is organized by anatomical architecture and by cellular interactions.
- The architecture of the system brings select immune cells together in discrete space and time and the interactions between the now adjacent T cells create the integrated dynamic response of the system.

(6) Maturation module: it is discussed before on maturation phase.

(7) Replication (proliferation) module: it takes place when a successful ISR results in the proliferation of B cells that have high affinities for the foreign pathogens that caused the response [39]. Proliferation and differentiation process of T cell and B cell acquires initiation of effectors function [5]. On the other hand, macrophages with an engulfed virus stimulate an increase in the proliferation of both helper and killer T cells, which are the key players in cell-mediated immunity and destroy virus infected tissue and cells to prevent any further spreading of the virus [25].

(8) Self-cleaning and tolerance module: phagocytes engulf the body of worn-out cells, other debris and activate the adaptive immune system [43]. More details are discussed before on maturation phase.

(9) Positive power module: it is a positive feedback that effectors helper is acting as process in which the effects of a small disturbance on a system include an increase in the magnitude of the perturbation of the system [44]. When cells start executing an action, they need pulses of positive power to be able to do the required action. The amount of positive power granted for the cell is determined by effectors rates. Immune system has a feedback loop influencing its own rate of change; such feedback can be direct or indirect.

(10) Negative power module: it is negative feedback helper effectors as opposite to positive feedback. A key feature of positive and negative feedback is thus that small disturbances get bigger, for example, cell inhibitors receptors.

### 3.7. Complex Adaptive Architecture

**Figure 7:**
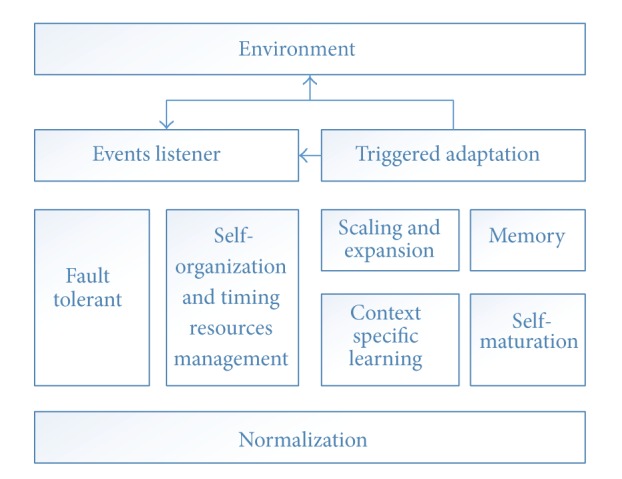
Complex adaptive architecture.

Human immune system has motivated scientists and engineers for finding powerful information processing algorithms that have solved complex engineering tasks. The following components are introduced (see Figure 7).

(1) Normalization: it is the initial process responsible for dividing large and complex data inputs into smaller and simple form for enhancing data processing and recognition.

(2) Self-mutation: data normalization enhances system capabilities on matching and recognizes new forms of data inputs and system detectors.

(3) Context specific learning: this process is responsible for tightly coupled integration module, since each domain has its own features and properties. The process contains self-learning service, which is critical to support adaptive features.

(4) Memory: memory is the storage and logging media which store (log) current and previous context state actions and feedback for future use.

(5) Scaling and expansion: immune system needs to scale actions, feedback, and effectors with certain rates based on triggered adaptation and context state.

(6) Triggered adaptation: adaptation is the final process before functions effectors perform the selected action. For example, adaptation process based on historical learning and the current context of the environment has been logged and actions are executed and then get the environment feedback.

(7) Fault tolerance: healthy system grants tolerance on the following levels: crash (the component either completely stops operating or never returns to a valid state), omission (the component completely fails to perform its service), timing (the component does not complete its service on time), and faults of an arbitrary nature, and finally it logs all failure events for further investigation and learning.

(8) Self-organization and timing resource management: these processes exist to govern allocation and deal with location of resources as required in the context [32], it orchestrate the actions, environment feedback, and increasing or decreasing system defectors with real time manner is a critical mission should be accomplished by complex adaptive system.

(9) There are a number of challenges in order to make complex adaptive system as mainstream computational science technology including the following.

- Performance of parallelization technology is one [45].
- Verification and validation of large scale models are another [45].
- Highly distributed and adaptive systems are too complex to predict the output and to debug.
- Some of ISR are not well understood yet.
- Biological systems are hierarchical involving several layers of complex interactions from basic chemical reactions to patient scale effects. The interaction between objects on a given level can be decomposable, so the interactions between objects on different levels are extremely sophisticated and complex [46].
- Any biological simulation for a poorly understood adaptive system with numerous levels of feedback from many sources is a complex task [46].

## 4. HCV Case Study

The immune system typically protects against infection and kills viruses; it is unusual that it is unable to clear the hepatitis C virus (HCV) when infected. Most people infected with HCV experience persistent infection whereby the virus evades, subverts, and/or weakens the immune system and survives for the life of the infected person. Such chronic infection with HCV often results in liver damage, which can lead to cirrhosis liver failure, liver cancer, and/or premature death [23]. Recently it has been demonstrated that the immune system plays an essential role in cancer dynamics [1]. The case study discuss immune system response in HCV; from the paper point of view and focuses on ISR phases with a solid biological definitions of IS operations consequences processes, ISR phases of HCV infection.

(1) Maturation: matured dendritic cells (mDCs) activate CD4+ and NK cells. CD4+ cells produce cytokines, such as IFN-g, that induce cytotoxic T lymphocytes (CTLs). CTLs can control replication by direct lysis of infected cells and also through production of cytokines that can inhibit viral replication [23].

(2) Recognition: after HCV infection there is an activation of NK cells, as well as processing of viral antigens by immature dendritic cells. From the other side, failure to clear HCV infection is due to a failure to initiate ISR at the appropriate time. Possible mechanisms of chronicity in HCV include failure of NK cells, dendritic cells, and CD4+ cells. This results in inappropriate or ineffective cytokine production that fails to control virus [47]. In vitro studies show that NK cells of healthy individuals can be inhibited by high concentrations of the HCV, and that NK cells of HCV infected individuals are altered in their cytokine production; and their capacity to activate NK cells, integrate signals fromarrays of activating, and inhibitory receptors [23].

1. HCV prevents MHC I molecules from appearing at the cell surface, so inhibitory receptors will not be engaged, so they almost evaded from ISR [48]. Nevertheless, NK's primary concern is the presence of MHC I molecules; if not, then something is wrong with that T cell and it should be destroyed [23].
2. Dendritic cells possess pattern recognition receptor (PRR) named toll-like receptor-3 (TLR3) which senses HCV infection leading to activation and production of numerous chemokines and inflammatory cytokines, macrophage inflammatory protein, and interleukin-6. While sensing RNA viruses is still unclear, they are primarily thought to be activated by intracellular stress signals, for example, damage associated molecular patterns [49], which is a complaint with danger theory. The chemokine/cytokine induction occurred late in HCV infection and was abrogated. Therefore, HCV was ultraviolet inactivated before infection indicating a dependence on the cellular recognition of HCV replication products [50].
3. During the early phase of HCV infection, large amounts of type I interferons (IFN-*α* and IFN-*β*) may be produced by HCV infected hepatocytes as well as dendritic cells to control viral replication. Dendritic cells produce high amounts of cytokines, such as IL-12 which has been shown to play an important role in stimulating IFN-*γ* production from activated T cells inducing the development of type Th1 used to activate ISR. Indeed, a recent study showed that an increased number of dendritic cells during acute HCV infection may be associated with viral clearance [49].
4. Viral proteins are chopped into small fragments (normalized) by APCs and then transported back to the cell surface where they are firmly held and paraded by dedicated molecular scaffolds called antigen receptors; these bits of viral protein are closely inspected by lymphocytes, some of which will recognize their presence, become activated, and embark upon an attempt to rid the body of the virus [23].

(3) Decision making: regulating immune attack by dictating the type of local forces deployed dendritic cells and also activated NK cells. Dendritic cells continuously express high levels of costimulatory molecules, which are inserted into their cell membranes and costimulatory molecules activate naive T cells [23]. Cytokines released following TLR binding by viral RNA include IFN-*α*, the same chemical we used to treat HCV, together with a variety of interleukins that stimulate activation of effectors lymphocytes including NK and T cells [23].

HCV specific memory cells are able to respond to naturally processed antigens. Nevertheless interferons can be effective inhibitors of viral replication and can suppress HCV in many people when used therapeutically [23].

(4) Execution: the effectiveness of T cell vaccine will depend on efficient induction and maintenance of an adequate repertoire of HCV specific memory cells [23]. Immune signals are sharing a common signaling pathway and propagate to amplify effectors cells; for example, TLR signals are propagated to tumor necrosis factor (TNF) receptor associated factor-6 for activation of NF-kB and mitogen activated protein kinases [49].

Inflammatory cytokine responses and TLR3-mediated chemokine play an important role in host immune response to HCV and the pathogenesis of HCV associated liver diseases [50]. Dendritic cells' stimulation produces high amount of IFN-*γ* which subsequently activates hepatic macrophages to enhance local inflammation, and TRL3 sensing plays a critical role in promoting liver inflammation [49].

Successful clearance of HCV infection requires the coordinated action of innate immunity and acquired immunity. After infection there is activation of NK cells, which cause infected cell to commit a form of ritual suicide called apoptosis as well as processing of viral antigens by dendritic cells [23].

## 5. Conclusions

The natural immune response has been subject to in-depth analysis of its processes, so we claim from high level point of view that immune system is a cognitive intelligent system, which utilizes its intelligent accumulative capabilities to turn the body from unhealthy state to healthy state. The paper focuses on the big picture of the cognitive ability of immune system response, a deep investigation carried out analyzing diversity, distributed computation, fault tolerance, dynamic learning, and adaptation and self-monitoring [6, 11] from biological operations perspectives to extract the computational model of natural biological system, which advances and enhances the cognitive science in general and in particular intelligent agent. The cognitive computational model presented by the paper attempts to be as a general framework for complex distributed adaptive system and their consequences disciplines. Behavior driven classification for immune system response was proposed. Maturation, recognition, decision making, and execution phases were discussed in detail from biological operations and components architecture modeling perspectives to identify the functions input, processing, and output of certain behavior. Then a hybrid architect for intelligent agent system inspired by immune system response was proposed. The computational model is based on services, helpers, and core system modules; core modules are the controllers of the adaptive workflow and system processes. By the end, a case study on HCV was discussed from the paper point of view for immune system response when invaded by HCV.

## 6. Future Work

Building a cognitive intelligent agent framework, with adaptive workflow ability for executing system consequences as mentioned on the proposed computational model; and that framework should influenced by study of the nervous system, and immunology “neuroimmunology” from a cognitive perspectives, as an integrated model.

Interdisciplinary nature of the framework needs further research on how to adapt and integrate natural biological system with computer science. The impact of successful implementation will serve a range of disciplines including but not limited to immune system simulation, drugs development, classification, outstanding problems' optimization tasks, machine learning, self-maturation, computer security, industrial applications, and military defense system as well as applying the framework on HCV as a simulation for immune system response when encountering HCV attack.

Multiprogramming paradigms are supposed be used on that framework including dynamic, functional, behavior driven, and agent-oriented; in order to sustain the above statement, further research is necessary on how the nervous system works and its direct effect on our immune system response. Furthermore, the aim is to build an integrated computational model for a cognitive compliant biology systems; that encompasses our holistic human well-being.
